# Development of a Sustainable, Simple, and Robust Method for Efficient l-DOPA Extraction

**DOI:** 10.3390/molecules24122325

**Published:** 2019-06-24

**Authors:** Katarzyna Polanowska, Rafal M. Łukasik, Maciej Kuligowski, Jacek Nowak

**Affiliations:** 1 Institute of Food Technology of Plant Origin, Faculty of Food Science and Nutrition, Poznań University of Life Sciences, 31 Wojska Polskiego street, 60-637 Poznań, Poland; maciej.kuligowski@up.poznan.pl (M.K.); jacek.nowaktz@up.poznan.pl (J.N.); 2 Laboratório Nacional de Energia e Geologia, I.P., Unidade de Bioenergia, Estrada do Paço do Lumiar 22, 1649-038 Lisboa, Portugal; rafal.lukasik@lneg.pt

**Keywords:** l-DOPA, *Vicia faba*, extraction, Parkinson’s disease, stability

## Abstract

l-3,4-dihydroxyphenylalanine (l-DOPA) is a medically relevant compound in Parkinson’s disease therapy. Several extraction methods of l-DOPA from beans, including velvet and faba beans, have been described in the literature. However, these methods require the use of strong acids, long extraction times, or complex downstream processing, which makes the extraction of l-DOPA expensive and energy-demanding, limiting its industrial application. In addition, the stability of l-DOPA during the extraction process is critical, further complicating the extraction of adequate amounts of this amino acid. This work is the first report on a simple, rapid, greener, and robust extraction method of l-DOPA. The developed method consists of a quick homogenization step followed by a double extraction with 0.2% *v*/*v* acetic acid for 20 min and was applied to faba bean at a ratio of 1:25 with respect to the extracting solvent. This study also investigated the stability of l-DOPA during extraction and thermal treatment. The proposed method demonstrated to be robust and extraordinarily efficient for numerous cultivars of faba bean, velvet bean, and food products containing faba beans.

## 1. Introduction

Nowadays, our society is concerned about the use of environmentally friendly products in everyday life. This leads people to choose more sustainable and renewable resources and products, including cosmetics, drugs, and products of natural origin. This social demand stimulates the search for more robust and at the same time more sustainable methods for the extraction of diverse high-value substances. l-DOPA is an amino acid commonly used in the treatment of Parkinson’s disease [1]. Parkinson’s disease affects mainly the motor function as a consequence of the degeneration of dopaminergic neurons in specific brain areas. Since dopamine is not able to cross the blood–brain barrier, Parkinson’s disease patients are treated with its precursor, l-DOPA [2]. Up to now, the use of l-DOPA has been considered as the best treatment for Parkinson’s disease; however, l-DOPA administration can cause many side effects such gastrointestinal disturbances or dyskinesias [1,3].

l-DOPA is found in several natural products, such as faba beans (*Vicia faba*) and velvet beans (*Mucuna pruriens*) [4,5]. Velvet bean is considered the richest source of l-DOPA, as it can contain even as much as 9% wt of l-DOPA [3]. Although velvet bean is very rich in dopamine precursor, its use as a source of l-DOPA is rather limited by its restricted geographical distribution in the tropical regions of Africa and Asia. An alternative source of l-DOPA is faba bean, whose annual production is almost 4.5 Mtonnes, predominantly in South America, Asia, and Europe [6]. The wider geographical spread of faba makes this plant a potentially more interesting source of l-DOPA than velvet bean.

Regardless of the source of l-DOPA, there is a strong need for more sustainable, environmentally friendly, and as little as possible detrimental methods of l-DOPA extraction. Although several extraction methods have been presented in the literature, they employ mineral or organic concentrated acids or alcohols [5,7,8,9]. The use of mineral or organic concentrated acids for l-DOPA extraction, though efficient, is limited by the requirement of costly and energy-demanding downstream processes. Also, the potential degradation of l-DOPA in a strongly acidic environment is a serious constraint for the prospective application of the obtained l-DOPA. The use of alcohols, e.g., methanol or ethanol, can be an alternative, but the process of alcohol removal may affect l-DOPA concentration in the final extracts [4].

This work proposes a simple, sustainable, and robust method for l-DOPA extraction from faba beans. Several extraction parameters, including the use of solvents respectful of the environment and of human health, extraction time, pre-processing of raw material, number of extraction runs, and others factors were analyzed to determine the best extraction conditions to achieve the maximum l-DOPA concentration. The efficiency of the proposed method was validated by testing it in l-DOPA extraction from velvet beans. Finally, the robustness of the extraction process was tested on 10 other cultivars of faba beans and six food products containing faba beans. In addition, the influence of food processing steps on the l-DOPA stability was analyzed.

## 2. Results and Discussion

### 2.1. Optimization of the Extraction Conditions

**Figure 1:**
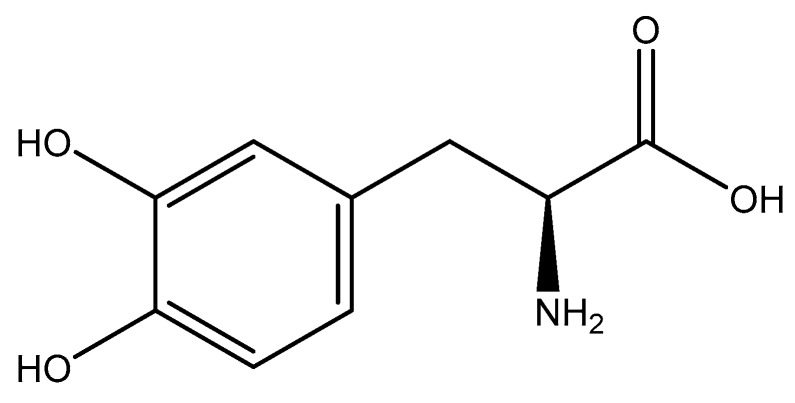
The chemical structure of l-3,4-dihydroxyphenylalanine (l-DOPA).

**Figure 2:**
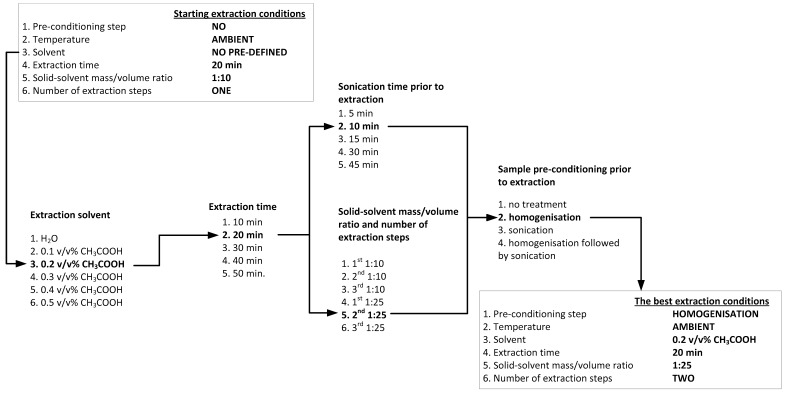
The decision tree employed to determine the best l-DOPA extraction conditions from *Vicia faba* var. *major* “Bachus”.

The l-DOPA (Figure 1) extraction procedure was optimized using dry beans of *V. faba* var. *major* ‘Bachus’. The sequence of analysis of the examined parameters presented in Figure 2 and the tested conditions for each parameter were selected arbitrary.

#### 2.1.1. Extraction Solvent

The selection of the extraction solvent was made by testing the extraction of l-DOPA from 1 g of faba beans with 10 mL of extraction solvent for 20 min. For these trials, water and acetic acid at five different concentrations, namely, 0.1, 0.2, 0.3, 0.4 and 0.5% *v*/*v*, were tested. As shown in Table 1, acetic acid was three-fold more efficient for the extraction of l-DOPA than water. Even as little as 0.2% *v*/*v* acetic acid allowed increased l-DOPA concentration in the extract from 25.2 ± 1.1 to 77.5 ± 1.5 μg/g dry weight (dw) of faba beans. A further increase of the extraction solvent acidity, i.e., the use of a 0.4 or 0.5% *v*/*v* acetic acid solution, reduced l-DOPA content to as low as 62.2 ± 0.8 μg/g dw of faba beans.

**Figure 3:**
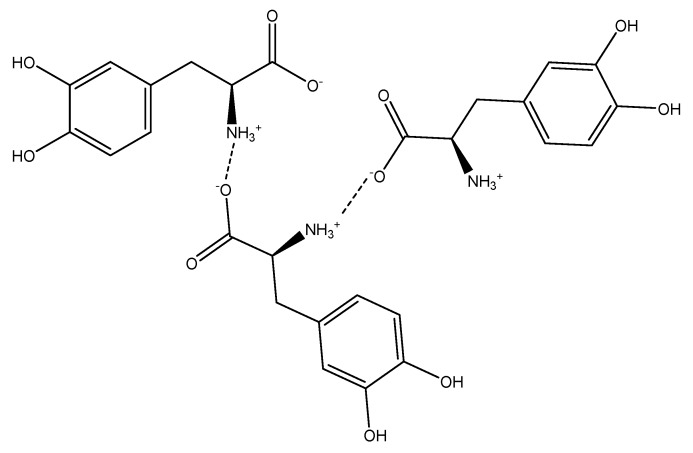
Example of the potential l-DOPA intermolecular network formed during extraction t a neutral pH.

When analyzing the obtained results, it is clear that aqueous acetic acid is a better solvent than water for the extraction of l-DOPA from faba beans. One of the reasons might be l-DOPA dissociation in acidic conditions. At a concentration of acetic acid above 0.1% *v*/*v*, when acetic acid is present in the solution in a dissociated form (pKa of acetic acid is 4.76) [10], proton concentration is relatively high, and both amine and carboxylic acid groups of L-DOPA are protonated. The resulting positively charged molecule is stable in the acetic acid solution. The same does not occur in the case of water extraction, because at the almost neutral pH of water containing faba bean, which is 6.20, l-DOPA is dissociated and forms a network of intermolecular bonds involving the protonated amine groups ($–NH_{3}^{+}$) and the deprotonated carboxylic acid groups ($–COO^{-}$), as shown in Figure 3. Such a network is less soluble in water, hence, l-DOPA concentration in water used as the extraction solvent is lower than that in an acetic acid solution.

Another reason for the more efficient extraction of l-DOPA by acetic acid might be that even in a slightly acidic environment as that of 0.2% *v*/*v* acetic acid, the polysaccharide matrix of faba bean can be partially hydrolyzed. The performed analyses showed that acetic acid improved pentosan hydrolysis by 20.5% in comparison to water-only extraction. Since pentosans constitute up to 21.3% of all non-starch polysaccharides [11] in faba bean, the hydrolysis of these polysaccharides may physically enhance the extractability of l-DOPA, promoting its release.

By analyzing the results of this study for different concentrations of acetic acid, it is clear that a concentration of acetic acid as high as 0.2% *v*/*v* was sufficient to maximise l-DOPA extraction. A further increase of acetic acid concentration had no statistically significant influence on the extraction efficiency (0.2% *v*/*v* versus 0.3% *v*/*v* acetic acid) or even reduced l-DOPA content, as seen for more highly concentrated solutions of acetic acid. Therefore, a 0.2% *v*/*v* solution of acetic acid was selected as the best solvent for the extraction of l-DOPA.

One of the conclusions coming from the literature is that there are no established extraction method and solvent for l-DOPA extraction from faba beans. For example, Cardador-Martinez et al. (2012) used 0.83 M perchloric acid for l-DOPA extraction and obtained at least 280 µg/g dw of faba beans [9]. When less concentrated perchloric acid (0.2 M) was employed, only 40 µg of l-DOPA per 1 g dw of faba beans was extracted [5]. In experiments using 1% *v*/*v* of formic acid as the extraction solvent for 42 types of genotypes of faba beans, the concentration of l-DOPA varied from 0.09 to 1.1 mg/g dw [7].

Therefore, it can be concluded that when either mineral or organic acids were used, the achieved l-DOPA concentration was in the same range as that obtained in this work. However, it is important to underline that the acids used in the literature [5,7,9] were in general more concentrated than the acetic acid tested herein. Hence, their use might be considered as less convenient because of the considerable risk of corrosion of the extraction equipment and of the requirement of further processing, which could definitively reduce l-DOPA concentration and increase the operation costs of its production.

An alternative extraction method to those based on acids involves the use of alcohols (methanol or ethanol). A study employing a two-stage extraction method, using water in the first stage and an 80% methanol aqueous solution in the second step, 2.03 mg of l-DOPA/g dw was extracted from faba beans [12]. Etemadi et al. (2018) used a 95% ethanol solution and obtained an extraordinarily high amount of l-DOPA (7.2 mg/g dw of faba beans) [8]. However, taking into account that l-DOPA is practically insoluble in ethanol and only partially soluble in water (3.3 g/L) [13], the results presented by Etemadi et al. (2018) are rather questionable, because the claimed l-DOPA concentrations obtained exceed the l-DOPA solubility limit. Therefore, these results should be taken with some precaution.

Although methanol and ethanol seem to be better for l-DOPA extraction, their use requires evaporation after the extraction. Therefore, extractions methods based on alcohols are more energy- and cost-demanding than that using 0.2% *v*/*v* acetic acid.

#### 2.1.2. Extraction Time

The effect of the extraction time on l-DOPA concentration was examined. As it could be expected, time should have a positive effect on the extraction efficiency. Indeed, this was observed for extraction times up to 20 min; however, a further increase of the extraction time demonstrated an opposite tendency, as can be observed in Table 2. An increase in the extraction time above 20 min had a clear negative effect on l-DOPA content in the liquid extract. l-DOPA concentration was reduced from 77.5 ± 1.5 to 71.9 ± 0.7 µg/g dw when increasing the extraction time from 20 to 60 min. Although these results may seem surprising, it is important to consider that the decreased l-DOPA concentration might reflect the moderate stability of this amino acid. An increase in the extraction time can promote l-DOPA degradation to dopachrome or dopaquinon, molecules responsible for oxidative stress and selectively cytotoxic for neuronal cells in patients suffering from Parkinson’s disease [14]. Consequently, such degradation would lower l-DOPA content in comparison to the l-DOPA amounts obtained for shorter extraction times, especially, 20 min. In the literature, we did not find any reports on the influence of the extraction time on l-DOPA content in the obtained extracts. However, in general, all extractions reported were performed with prolonged extraction times, even as long as 3 days [8,15]. For example, Pulikkalpura et al. (2015) reported that during a multistage extraction lasting for 24 h, with an equivolume formic acid/ethanol mixture, l-DOPA from *M. pruriens* seeds underwent a significant degradation [4]. They found a reduction of l-DOPA content by as much as 52.11%. Therefore, it is clear that a prolonged extraction time might negatively influence l-DOPA stability and consequently its content in the final extract.

#### 2.1.3. Sonication Time Prior to Extraction and Number of Extraction Runs with Different Solid/Solvent Ratios

The influence of sonication treatments of 5, 10, 15, 30, or 45 min prior to extraction and the number of extraction runs (one, two, or three) with different solid- (faba beans) to-solvent (0.2% *v*/*v* acetic acid) ratios of 1:10 and 1:25 was examined.

It can be expected that the application of cavitation forces to the system should increase l-DOPA concentration in the final extract. Indeed, a positive effect of ultrasounds employment was observed up to 10 min of treatment; however, a further increase of the sonication time decreased l-DOPA content in the extract. More specifically, for 10 min of sonication, 80.6 ± 0.5 μg/g dw was obtained, whereas for 15 min, l-DOPA content was only 72.6 ± 3.8 and dropped further to 67.6 ± 1.8 μg/g dw for 45 min of sonication. A possible explanation of these results might be that sonication caused thermal degradation of l-DOPA. This effect is similar to that observed for prolonged extraction times, discussed above. Dhanani et al. (2015) studied the influence of sonication time on l-DOPA extraction yield. They subjected velvet beans seeds to sonication up to 15 min [16]. In general, l-DOPA content increased with the increase of the sonication time for most of the varieties, however, for one of them, l-DOPA content peaked after 10 min of sonication and decreased after longer sonication times. Therefore, for even longer sonication times, e.g., 30 min, l-DOPA concentration might further decrease, supporting the results presented in this work.

**Figure 4:**
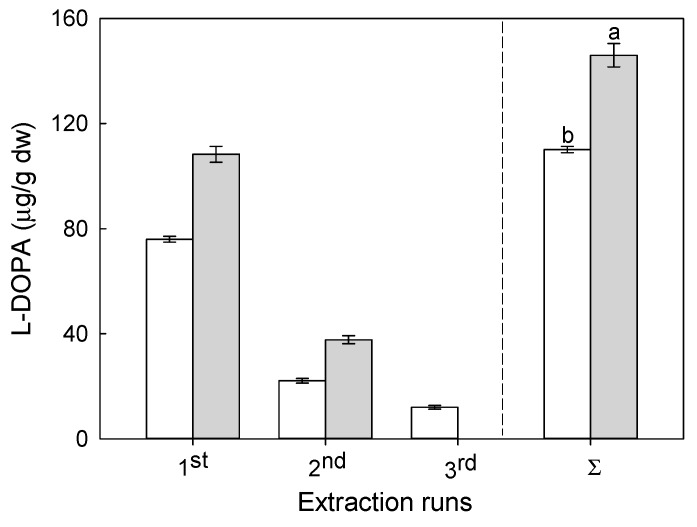
l-DOPA concentration as a function of the extraction runs for 1:10 (white bars) and 1:25 (grey bars) solid mass/solvent volume ratio. The total l-DOPA amounts obtained after three extraction runs for both systems is given for comparison purposes. Different letters next to l-DOPA content indicates statistically significants differences according to the Tukey’s test (*p* > 0.05).

Another independent variable studied was the number of extraction runs with different faba beans-to-acetic acid ratios. As it can expected, both the number of extraction runs and the different faba beans-to-extraction solvent ratios should affect positively l-DOPA concentration in the extract. As depicted in Figure 4, either an increase in the number of extraction runs or the use of a more diluted system, i.e., a lower solid-to-solvent ratio, increased l-DOPA concentration in the extract.

At first glance, it can be concluded that in the first extraction run, a vast part of l-DOPA was extracted and, not surprisingly, the amount of l-DOPA was higher for lower solid/solvent ratios. For the 1:10 (m/v) faba bean/acetic acid ratio, the amount of l-DOPA was 76.0 ± 1.1 μg/g dw of faba beans, while for a 2.5-fold higher volume of solvent, a substantial increase of l-DOPA, corresponding to 43%, was observed. Interestingly, a similar trend was not observed in the second extraction run, which yielded 22.1 ± 0.9 and 37.7 ± 1.5 μg of l-DOPA/g dw for 1:10 and 1:25 (m/v) solid/solvent ratios, respectively. Further extraction runs demonstrated that only for thd 1:10 (m/v) solid/solvent ratio additional 12.0 ± 0.7 μg of l-DOPA/g dw was obtained. Analyzing the literature data, most of l-DOPA extraction methods from faba bean used only a single extraction step with solid/solvent ratios varying from 1:5 to 1:100 [5,7,8,9]. However, none of those works compared different solid/solvent ratios for the same extraction methodology. Hence, it is not possible to draw a clear conclusion for l-DOPA. On the other hand, considering the extraction of phenolic compounds from a natural matrix such as grape seeds, an increase in the solvent-to-solid ratio improved the extraction efficiency. For example, Casazza et al. (2011) found that for extraction times of 9, 19, and 29 h, the increase of the solvent from 3.3 to 10 in relation to grape seeds amount enhanced the extraction yield of a wide variety of phenolic compounds [17].

In our experiments, the total amount of l-DOPA extracted in three runs using a 1:25 (m/v) faba bean/acetic acid ratio was 33% higher than that obtained for a 1:10 (m/v) faba bean/acetic acid ratio. Therefore, although the third extraction promoted a further extraction of l-DOPA, the solid-to-solvent ratio considerably affected l-DOPA yield. Thus, it is clear that other factors exist, which hamper the extraction of l-DOPA from faba beans. To clarify this phenomenon, pre-conditioning of samples prior to extractions was studied.

#### 2.1.4. Sample Pre-Conditioning

To identify the factors that could affect l-DOPA extraction yield, the pre-conditioning steps prior to extraction were examined. Among the analyzed steps were homogenization and sonication, considered individually as well as sequentially, with homogenisation preceding sonication. The obtained results are presented in Table 3. Although the sonication effect on l-DOPA extraction from faba beans was already described in the previous section, the 10 min treatment was examined again because of the double extraction run used at this stage.

The results obtained for different pre-conditioning conditions showed that homogenization increased l-DOPA concentration in the extract with respect to the extract obtained without any pre-conditioning. l-DOPA concentration in the extract without pre-conditioning was 146.0 ± 4.5 μg/g dw, whereas l-DOPA concentration after homogenization reached 151.5 ± 5.1 μg/g dw. Two other approaches, namely, sonication and homogenization preceded by sonication, caused a drop of l-DOPA concentration to a value lower than that obtained for the sample without any pre-conditioning. Therefore, it can be concluded that sonication was confirmed to have a considerable negative effect on the final l-DOPA concentration because, either alone or in combination with homogenization, significantly reduced l-DOPA concentration to a value as low as 124.2 ± 2.1 μg/g dw. These results support previous conclusions that a prolonged extraction time and the thermal effects of sonication have a negative effect on l-DOPA stability. This, in turn, can be explained by l-DOPA susceptibility to oxidation when exposed to the temperature or air [18,19].

### 2.2. Validation of the Best L-DOPA Extraction Conditions

The best conditions for l-DOPA extraction from dry beans of *V. faba* var. *major* “Bachus” involved the homogenization of the sample prior to extraction performed at room temperature with 0.2% *v*/*v* acetic acid and a dry bean/solvent ratio of 1:25 (m/v), using two consecutive extraction runs lasting 20 min each. These conditions were determined for the aforementioned cultivar of faba bean and were employed for the extraction of velvet bean, to verify the efficiency of our method. Velvet bean is a legume characterized by a very high content of l-DOPA [4]. Depending on the variety of velvet bean, l-DOPA contents are in the range from 3.6 to 9.1 g/100 g dw [3]. Therefore, this bean can be considered an ideal reference system for testing the efficiency of the described l-DOPA extraction method.

The application of the extraction procedure allowed achieving an l-DOPA content as high as 50.7 ± 0.6 mg/g dw. This result is in great agreement with those presented in the literature, indicating the achievement of an l-DOPA concentration of 5 g/100 g dw [19,20]. Therefore, it can be concluded that the proposed procedure is adequate for l-DOPA extraction fromfaba beans, and the results obtained are comparable to those presented in the literature [19,20].

### 2.3. L-DOPA Extraction from Dry Faba Beans

The extraction processes employed for velvet beans allowed to confirm the presence of a high content of the amino acid in the analyzed feedstock. Hence, the best conditions determined for dry beans of *V. faba* var. *major* ‘Bachus’ were used to extract l-DOPA from four other cultivars of faba beans *V. faba* var. *major* (M) and six cultivars of *V. faba* var. *minor* (m). The obtained results are presented in Table 4. Analyzing these data, it can be concluded that, independently of the cultivar, *V. faba* var. *major* contained much less l-DOPA than *V. faba* var. *minor*. The difference observed was even 7.5-fold, i.e., 151.5 ± 5.1 versus 1152.0 ± 17.6 μg/g dw for Bachus (M) and Fernando (m), respectively. Also, the highest l-DOPA content found in the cultivar of *V. faba* var. *major* was lower than the lowest l-DOPA content found among all cultivars of *V. faba* var. *minor*. For example, Bolero (M) was found to contain l-DOPA at a concentration of 335.8 ± 3.2 µg/g dw, while Albus cultivar contained 382.2 ± 4.0 µg of l-DOPA per each g of dry weight of faba beans.

Another interesting observation is that among all studied cultivars, larger differences in l-DOPA concentration were found for *V. faba* var. *minor*. For those cultivars, the l-DOPA content could be as high as 1152.0 ± 17.6 μg/g dw or as low as 382.2 ± 4.0 μg/g dw, corresponding to almost an 800 μg/g dw difference. For *V. faba* var. *major*, the differences were lower, reaching no more than ~200 μg/g dw.

In spite of being a precursor of many alkaloids, catecholamines, and melanin, l-DOPA plays an important role in the chemical defence against herbivores and competing plants [21,22]. The literature reports that the l-DOPA content increases during the germination of faba beans but also as a response to stressing agents such as phytochemical and peptide elicitors (fish protein hydrolysates, oregano extract, lactoferrin), bacterial elicitors, and L-ascorbic acid [23,24,25]. In addition, the cultivation conditions, namely, climate and soil fertility, may markedly influence l-DOPA content in plants [4]. However, the differences in l-DOPA content are primarily genetically determined. The study of 42 different genotypes of faba bean demonstrated substantial differences in l-DOPA concentration which varied from 90 µg/g dw to even 1100 µg/g dw [7], i.e., in exactly the same range as reported herein.

### 2.4. L-DOPA Extraction from Food Products

The results obtained for dry beans prompted us to study l-DOPA content in food products. Among the tested products, three frozen and three canned faba beans were analyzed, and the obtained results are presented in Table 5. As it can be observed, in general, frozen products contained more l-DOPA than canned products; however, the differences were not substantial and mostly depended on the brand of the product. Unfortunately, the examined food products lacked specific information about the cultivar used, therefore, it was impossible to compare their quality. Nevertheless, one of the reasons for the slightly lower content of l-DOPA extracted from canned foods might be the thermal treatment applied during the preparation of such products. As it was shown, long extraction times and sonication have a negative effect of the stability of l-DOPA, therefore, processing faba beans at high temperature might have a clear negative effect on l-DOPA content.

To examine this effect, the frozen faba beans analyzed in this work were subjected to cooking, as described in the experimental section. The obtained results are presented in Table 5 and confirm our hypothesis. After cooking, the l-DOPA content of frozen food products dropped significantly in comparison to that in uncooked products. In one case (food product indicated as P2), no l-DOPA was found after processing. This confirms that the thermal processing of food has a strong negative effect on l-DOPA concentration in the final product and it explains the lower l-DOPA content in thermally processed canned faba beans compared to frozen ones.

Another relevant observation is that frozen food products contained a significantly lower amount of l-DOPA than the above-mentioned dry faba beans (Table 4). The smallest difference was still very high, corresponding almost to 40% for l-DOPA content in the frozen product (K1) with respect to l-DOPA content in Bachus (M). One reasons for such significant differences might be that in the course of the preparation of the frozen products, thermal pre-conditioning such as blanching is applied. This, in addition to inactivating enzymes, affects the l-DOPA content in the final product. Therefore, to validate our conclusions, l-DOPA stability was tested.

#### l-DOPA Stability

**Figure 5:**
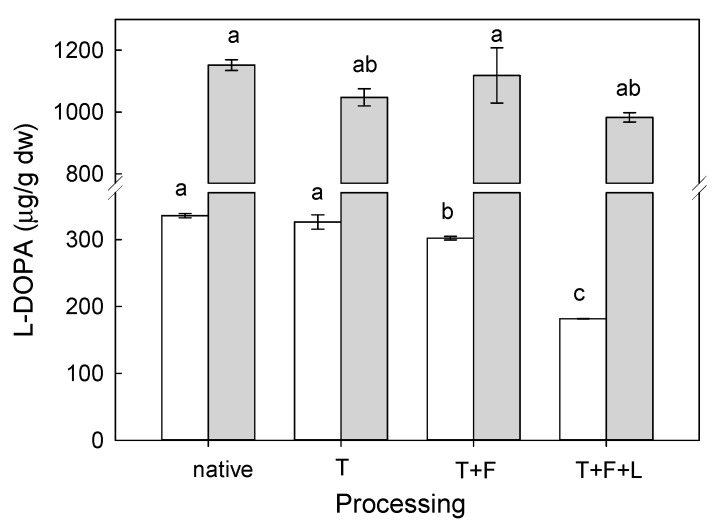
l-DOPA concentration found in *V. faba* var. *major* Bolero (white bars) and in *V. faba* var. *minor* Fernando (grey bars) after heating (T), freezing (F), lyophilisation (L); extraction was performed in the best conditions determined before. The same letter next to the l-DOPA contents indicates no statistical difference according to the Tukey’s test (*p* > 0.05).

Similar to either frozen or canned products of faba beans, the thermal stability of l-DOPA was tested also in dry faba beans. Two dry faba beans, Bolero (M) or Fernando (m) cultivars were tested. These faba beans were selected because their l-DOPA content is the highest among all *V. faba* var. *major* or *minor* analyzed in this study. Therefore, they seemed ideal to detect any influence of thermal processing by autoclaving followed by freezing and, finally, lyophilization. The obtained results are presented in Figure 5.

As shown in Figure 5, any kind of treatment reduced the l-DOPA content. This occurred for both *V. faba* var. *major* and *V. faba* var. *minor*. Among all processing steps examined, namely, heating, freezing, and lyophilisation, the last one affected l-DOPA content the most. For both varieties of faba bean, the highest drop in l-DOPA content was between 12% (Fernando) and 40% (Bolero) when compared to the l-DOPA content after heating or freezing. Also, the literature confirms l-DOPA instability due to thermal processing of faba beans (cooking, heating, freezing, or lyophilisation) presumably caused by oxidation [18]. For example, Gurumoorthi et al. (2008) found that boiling velvet beans decreased their l-DOPA content even by 27.8% when compared to uncooked velvet beans [26].

Therefore, the results presented here and those reported in the literature confirm the data obtained for food products presented above.

## 3. Materials and Methods

### 3.1. Plant Materials and Chemicals

The dry beans of six cultivars of *V. faba* var. *minor*, namely, “Albus”, ”Amigo”, ”Amulet”, “Fernando”, ”Granit”, ”Olga”, and of five cultivars of *V.faba* var. *major*, namely, ”Bachus”, ”Bolero”, ”Bonus”, “Rambo”, ”White Windsor”, used in this work were kindly provided by Hodowla Roślin Strzelce Sp. z o. o. Grupa IHAR and “Spójnia” Hodowla i Nasiennictwo Ogrodnicze Sp. z o.o (Poland).

Besides the cultivars mentioned above, also faba beans in food products were tested. For these trials, frozen faba beans of three brands, named K1, K2, and K3, as well as canned faba beans of three other brands, called P1, P2, and P3, were used. The K1, K2, K3 and P1, P2, P3 names were used to substitute the original product names to avoid advertizing. The names are known to the authors of this work.

To confirm the adequateness of the determined conditions for l-DOPA extraction, velvet beans (*M. pruriens*) as feedstock rich in l-DOPA [19] obtained from an internet shop (http://www.shamanshop.pl) were examined in this work too.

HPLC standards of l-DOPA (l-3,4-dihydroxyphenylalanine), whose chemical structure is depicted in Figure 1, and acetic acid (99.5%) employed for the extraction trials were bought from Sigma Chemical Co., (St. Louis, MO, USA). Distilled water of 18.2 MΩ/cm purity was produced by the Milli-Q system (Millipore, Bedford, MA, USA).

### 3.2. Determination of the Best Conditions for l-DOPA Extraction

A cultivar of *V. faba* var. *major* “Bachus” was selected arbitrarily to determine the best l-DOPA extraction conditions. Prior to extraction, dry beans of selected cultivars were ground using a laboratory grinder (WZ-1, ZBPP, Bydgoszcz) and sifted to remove particles with size smaller than 1 mm. Such prepared samples were stored at 4 °C in sealed containers prior to further work.

All l-DOPA extractions were performed at ambient temperature in 50 mL centrifuge tubes according to the following procedure. A known amount (1.0 g) of dry faba beans was placed in the centrifuge tube, and the exact amount of extraction solvent was added. Such prepared mixture was next placed in a roller agitator (MX-T6-S, DLab, Beijing, China) for a pre-defined extraction time. Once the extraction time elapsed, the sample was centrifuged for 10 min t 5096 g (MPW-350R, MPW Med. Instruments, Warsaw, Poland). In the following step, the obtained sample was filtered (paper filter, type 389, Alfachem, Lublin, Poland) to obtain a liquid that was collected in a volumetric flask for further analysis, as described in a subsequent section.

Figure 2 demonstrates the decision tree according to which the best conditions to achieve maximal l-DOPA concentration were determined. The determination of the best extraction conditions started with a single extraction run performed for 20 min with a 1:10 solid (dry beans)/solvent mass/volume ratio. These conditions were employed to pre-determine the best extraction solvents for l-DOPA extraction. For this purpose, water and acetic acid in the concentration range of 0.1–0.5% *v*/*v* were examined. The pH of such suspensions was determined using a pH meter (CP-411, Elmetron, Zabrze, Poland). In the next step, the best extraction time with the pre-selected solvent was determined. For this purpose, the extraction of l-DOPA for 10, 20, 30, 40, or 60 min was tested. Subsequently, the influence of ultrasound pre-conditioning before l-DOPA extraction as well as of different solid/solvent mass/volume ratios and number of extraction runs were tested. For this purpose, ultrasound-assisted extraction was performed using an ultrasound bath (bath power: 80W, continuous mode at the frequency of 40 kHz, Polsonic, Warsaw, Poland) for 5, 10, 15, 30, and 45 min at previously determined conditions (single extraction during 20 min with 0.2% *v*/*v* CH_3_COOH, 1:10 solid/solvent mass/volume ratio). The 1:10 and 1:25 solid/solvent mass/volume ratios were tested to establish the most suitable amount of solvent for the most efficient l-DOPA extraction. In addition, the same trials were also performed to determine the number of extraction runs. The determination of the best extraction conditions was followed by the analysis of the effect of different physical pre-conditioning steps of dry beans prior to l-DOPA extraction. For this, homogenization of ground dry beans for 30 sec at 20,000 rpm (H 500, PolEko Aparatura, Wodzisław Śląski, Poland) and subsequent sonication were tested. For comparison purposes, homogenization and sonication were applied separately to the dry beans inthe same conditions.

To validate the determined best conditions for l-DOPA extraction, the same procedure was employed for velvet bean (*M. pruriens*), a legume defined as one of the richest sources of l-DOPA [19]. The extraction conditions established above were used for 10 additional cultivars of faba beans (*V. faba* var. *major* and *V. faba* var. *minor*) listed in the plant materials and chemicals section.

### 3.3. Extraction of L-DOPA from Food

The previously determined conditions for l-DOPA extraction were employed for three kinds of frozen (K1, K2, K3) and canned (P2, P2, P3) faba beans products. Prior to extraction, the frozen faba beans were cooked for 15 min in boiling water (1:4 faba bean/water mass/volume ratio) according to the manufacturers’ recommendation listed on the packaging. Uncooked, cooked, and canned (brine was discarded) faba beans were immediately subjected to ^l-DOPA extraction according to the best methodology described above.

### 3.4. Influence of Thermal Pre-Conditioning on L-DOPA Stability

Thermal pre-conditioning of faba beans was tested to determine its influence on l-DOPA content after extraction. For this purpose, previously prepared powdered beans of *V. faba* var. *major* “Bolero” were mixed with distilled water in Erlenmeyer flasks (250 mL) in a 1:2.5 solid/water mass/volume ratio. The obtained mixtures were subjected to thermal heating at 121 °C for 15 min in an autoclave (ASV-E, SMS Sp. z o. o., Warsaw, Poland). After cooling down to room temperature, the samples were frozen at −80 °C for 24 h and next lyophilized at −72 °C for 65 h using a freeze drier (Christ, Alpha 2–4 LD plus, Osterode am Harz, Germany). The l-DOPA content in the samples was analyzed after each above-mentioned step. For l-DOPA extraction from each sample, the best conditions determined above were used. The l-DOPA content in native samples was also determined using the same extraction procedure.

### 3.5. Chemical Analysis

#### 3.5.1. Faba Bean Dry Mass Analysis

The dry matter content of all samples was determined in triplicates by oven-drying at 105 °C to a constant weight. The obtained results are given in Table 6.

#### 3.5.2. HPLC Analysis

The qualitative and quantitative analyses of l-DOPA in the liquid fractions obtained after extraction were performed using high-performance liquid chromatography (HPLC). All analyses were carried out using the Waters 2695 HPLC system equipped with a Waters 2996 photodiode array detector (Waters Associated, Milford, MA, USA). The analytical column was fitted with a reverse-phase Supelco Discovery C18, 250 mm × 4.6 mm, with a packing material of 5 µm particle size. The samples were eluted in an isocratic flow with a mobile phase consisting of 3% *v*/*v* methanol and 97% *v*/*v* of an aqueous solution of 0.2% *v*/*v* acetic acid at 1 mL/min flow rate. The qualitative analysis of l-DOPA was performed at 280 nm, and the quantitative determination was carried out by means of external calibration. All HPLC analyses were performed in triplicate.

### 3.6. Statistical Analysis

Statistically significant difference between the obtained results was determined using the Tukey’s multiple means comparison test with *p* > 0.05 set as the criterion of no significance. This analysis was carried out using Statistica 12 software (StatSoft, Inc., Tulsa, OK, USA).

### 3.7. Uncertainty Analysis

Standard uncertainty (*u*) was determined for all reactions performed in this study. Each weighing was performed with a *u*(m) of 0.1 g. The extraction time was determined with u(*t*) of 1 sec.

All errors related to the experiments described above depended on the calibration technique used for products quantification, and an arbitrary error of 10% of the measured value was established for all analyses.

## 4. Conclusions

This study presents a simple, efficient, and reliable extraction method of l-DOPA, a pharmacologically important compound present in faba beans. The developed method overcomes the common problems linked to l-DOPA stability contributing to the improvement of the process economics. The highest amino acid extraction efficiency was obtained with a process involving a rapid homogenization step preceding a double extraction with 0.2% *v*/*v* acetic acid for 20 min, using a faba bean-to-extracting solvent ratio of 1:25. l-DOPA was proved to be unstable when subjected to long extraction or any other pre-processing treatment. The developed method allowed to extract l-DOPA from various cultivars and allowed to demonstrate great differences in the amino acid content of the tested faba beans cultivars (151.5–1152.0 µg/g dw). Similarly, the studied food products containing faba bean were characterized by a significant variation in l-DOPA content; in addition, they contained a considerably lower amount of l-DOPA (0.0–92.3 µg/g dw) than dry beans. The method described in this work demonstrated to be robust and reliable for routine l-DOPA analyses, confirming its great potential for industrial applications.

## Figures and Tables

**Table 1:** Efficiency of l-DOPA extraction^A^ from dry beans of *V. faba* var. *major* “Bachus” depending on the solvent and pH of the obtained extracts.

Extraction Solvent	l-DOPA Content (µg/g dw)	pH
H_2_O	25.2 ± 1.1 ^d^	6.20
CH_3_COOH (0.1% *v*/*v*)	72.1 ± 2.1 ^b^	4.80
CH_3_COOH (0.2% *v*/*v*)	77.5 ± 1.5 ^a^	4.66
CH_3_COOH (0.3% *v*/*v*)	77.8 ± 0.6 ^a^	4.36
CH_3_COOH (0.4% *v*/*v*)	71.7 ± 3.8 ^b^	4.23
CH_3_COOH (0.5% *v*/*v*)	62.2 ± 0.8 ^c^	4.18

Dw: dry weight of faba bean; ^A^ extraction conditions: 10 mL of extraction solvent per 1 g of faba bean, 20 min of extraction time, single extraction run. The same letter next to the l-DOPA content indicates no statistical difference according to the Tukey’s test (*p* > 0.05).

**Table 2:** Influence of the extraction time on l-DOPA extraction^A^ from dry beans of *V. faba* var. *major* Bachus.

Time (min)	l-DOPA Content (µg/g dw)
10	74.8 ± 1.4 ^ab^
20	77.5 ± 1.5 ^a^
30	75.4 ± 1.2 ^ab^
40	74.2 ± 2.9 ^ab^
60	71.9 ± 0.7 ^b^

^A^ extraction conditions: 10 mL of CH_3_COOH (0.2% *v*/*v*) used as the extraction solvent per 1 g of dry faba beans, single extraction run. The same letter next to the l-DOPA contents indicates no statistical difference according to the Tukey’s test (*p* > 0.05).

**Table 3:** Influence of sample homogenization^A^ and sonication^B^ as well as of their combination, i.e., homogenisation followed by sonication, on the efficiency of subsequent l-DOPA extraction^C^ from dry beans of *V. faba* var. *major* “Bachus”.

Pre-Conditioning	L-DOPA Content (µg/g dw)
No pre-conditioning applied	146.0 ± 4.5 ^c^
Homogenisation	151.5 ± 5.1 ^a^
Sonication	135.7 ± 1.8 ^ab^
Homogenisation proceeded by sonication	124.2 ± 2.1 ^b^

^A^ homogenization conditions: 20,000 rpm, 30 s; ^B^ sonication conditions: 40 kHz 2 × 40 W, 10 min; ^c^ extraction conditions: 25 mL of CH_3_COOH (0.2% *v*/*v*) used as the extraction solvent per 1 g of faba bean, 20 min of extraction time, two extraction runs. The same letter next to the l-DOPA content indicates no statistical difference according to the Tukey’s test (*p* > 0.05).

**Table 4:** l-DOPA amounts obtained after extraction^A^ from dry beans of different varieties of *V. faba* var. *major* (M) and *V. faba* var. *minor* (m).

Variety	l-DOPA Content (µg/g dw)	Variety	l-DOPA Content (µg/g dw)
Bachus (M)	151.5 ± 5.1 ^j^	Amigo (m)	421.8 ± 4.1 ^d^
Bolero (M)	335.8 ± 3.2 ^f^	Olga (m)	516.8 ± 5.9 ^c^
White Windsor (M)	191.5 ± 6.8 ^h^	Granit (m)	517.6 ± 3.0 ^c^
Bonus (M)	170.7 ± 4.4 ^i^	Albus (m)	382.2 ± 4.0 ^e^
Rambo (M)	225.2 ± 3.4 ^g^	Fernando (m)	1152.0 ± 17.6 ^a^
		Amulet (m)	784.8 ± 7.9 ^b^

^A^ extraction conditions: 25 mL of CH_3_COOH (0.2% *v*/*v*) used as the extraction solvent per 1 g of faba bean, homogenization (20,000 rpm, 30 s), 20 min of extraction time, double extraction run. The same letter next to the l-DOPA contents indicate no statistical difference according to the Tukey’s test (*p* > 0.05).

**Table 5:** l-DOPA content in the food products obtained after extraction^A^.

Food Product	l-DOPA Content (µg/g dw)
Frozen	
K1	92.3 ± 5.3 ^a^
K1 (C)	6.3 ± 1.0 ^e^
K2	29.7 ± 0.5 ^b^
K2 (C)	0.0 ± 0.0 ^f^
K3	21.1 ± 1.5 ^cd^
K3 (C)	16.3 ± 0.9 ^d^
Canned	
P1	24.0 ± 2.2 ^bc^
P2	0.0 ± 0.0 ^f^
P3	18.0 ± 1.3 ^d^

C: cooked; ^A^ extraction conditions: 25 mL of CH_3_COOH (0.2% *v*/*v*) used as the extraction solvent per 1 g of faba bean, homogenization (20 000 rpm, 30 s), 20 min of extraction time, double extraction run. The same letter next to the l-DOPA contents indicate no statistical difference according to the Tukey’s test (*p* > 0.05).

**Table 6:** Water content of materials examined in this work.

Material Name		Humidity (wt%)	Material Name		Humidity (wt%)
*V. faba* var. *minor*			Frozen food		
	Albus	13.37		K1	66.02
	Amigo	12.77		K2	67.43
	Amulet	11.76		K3	66.07
	Fernando	12.57	Canned food		
	Granit	13.44		P1	84.94
	Olga	13.25		P2	85.73
*V. faba* var. *major*				P3	78.99
	Bachus	9.56	*V. faba* var. *major* cultivar Bolero		
	Bolero	8.11		autoclaved	69.49
	Bonus	10.34		frozen	69.91
	Rambo	9.36		lyophylised	0.63
	White Windsor	9.24	*V. faba* var. *minor* cultivar Fernando		
Velvet bean		8.05		autoclaved	71.02
				frozen	71.25
				lyophilised	0.64
